# Unilateral Pulmonary Fibrosis in Pregnancy: A Post-Tubercular Sequelae

**DOI:** 10.7759/cureus.11864

**Published:** 2020-12-03

**Authors:** Tanishq S Sharma, Shivang Amin, Shilpa Sapre, Palak Golani, Sheetalba Zala

**Affiliations:** 1 Medicine, Shree Krishna Hospital, Anand, IND; 2 Obstetrics and Gynaecology, Pramukhswami Medical College, Anand, IND

**Keywords:** : pregnancy, pulmonary fibrosis, multi-drug resistant tb, sequelae, maternal outcome, fetal outcome

## Abstract

Pulmonary tuberculosis (TB) is one of the top 10 leading causes of death in the world. Multi-drug resistant TB can lead to short-term and long-term sequelae causing clinical, psychosocial, and financial burden on the diseased. Pregnancy in a woman with compromised pulmonary function is a challenge for the treating obstetrician. A multidisciplinary approach involving a respiratory physician, pre-conceptional counseling, and delivery at a tertiary care center can reduce maternal morbidity and mortality. Compliance with anti-tubercular treatment with regular follow-up can minimize the long term effects of pulmonary TB.

We report a case of unilateral lung collapse due to multidrug-resistant pulmonary TB in pregnancy with good maternal and fetal outcomes.

## Introduction

Multi-drug resistance [1] tuberculosis (TB) is a form of TB wherein there is no response to the two most effective anti-tubercular medicines, isoniazid, and rifampicin. It can lead to short term and long term sequelae [2] like pleural thickening with fibrosis, bronchiectasis, aspergilloma, and bronchogenic carcinoma. Physiological changes in the respiratory system add a significant burden in patients with pulmonary fibrosis.

We report here a case of a pregnant woman with left-sided pulmonary fibrosis secondary to multi-drug resistant TB. There are a few case reports of pregnant women with unilateral pulmonary fibrosis due to pulmonary TB [3].

## Case presentation

A 24-year-old primigravida was referred at 36 weeks of gestation for institutional delivery. She had no complaints of bleeding or leaking per vaginum and had taken regular antenatal visits. She was asymptomatic at the time of her visit.

The patient gave a history of pulmonary TB in childhood for which she received anti-tubercular treatment. She defaulted to the first course of treatment but later when she again developed symptoms full anti-tubercular treatment was taken. On detailed evaluation and confirmation by acid-fast bacilli culture sensitivity, it was found that the patient had developed multi-drug resistant TB as she was a chronic defaulter. Pulmonary function tests done in 2010 reported severe obstructive respiratory disease and moderate restrictive respiratory disease. The patient was referred to a higher center for pneumectomy, however, she did not go forward with the operation. The patient did not come for follow-up in the chest medicine department for the last six years.

Her family and social history were unremarkable. Counseling regarding the mode of delivery and the need for intensive care was discussed with the patient and her relatives. 

On general examination, pulse was 90/min, blood pressure was 110/70mm of Hg, her SpO2 was 98%. No pallor, cyanosis, icterus, clubbing, or lymphadenopathy was seen. Examination of her respiratory system revealed dullness to percussion on the left side and on auscultation there were crackles and decreased air entry and vocal resonance over the left hemithorax. Despite these findings, the patient had no complaints of breathlessness or cough. The obstetric examination was normal and corresponding with the gestational age. Following lab investigations were additionally done which all ended up being within normal limits (Table 1).

**Table 1:** Laboratory Investigations on admission HB: haemoglobin; MCHC: mean corpuscular hemoglobin concentration; RDW: red cell distribution width.

Total Leukocyte count (x1000/ul)	Red blood cell count (million/cmm)	Haemoglobin (g/dl)	Haematocrit (%)	Mean corpuscular volume (fl)	Mean corpuscular HB(pg)	MCHC (g%)	RDW (fl)	Platelet count (x1000/ul)
11.7	4.43	12.3	36.6	82.6	27.8	33.6	37.8	272

**Figure 1:**
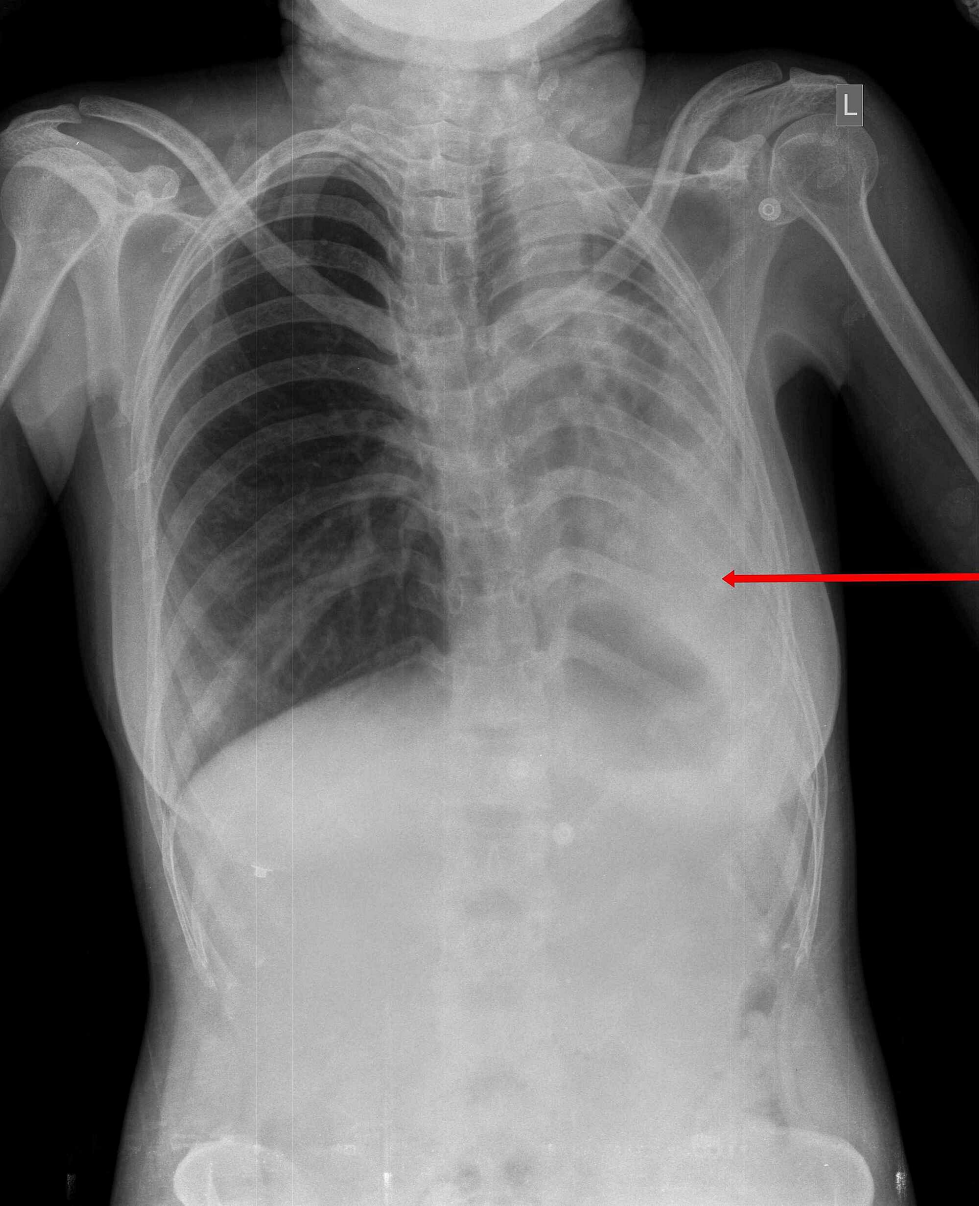
Chest radiograph, posteroanterior view, showing unilateral left side post tuberculosis fibrosis

On the radiograph of the chest with an abdominal shield taken in the posteroanterior view, there was evidence of diffuse radio-opacity involving left hemithorax with multiple small round to oval radiolucencies involving left mid-lower zones and mild emphysematous changes in the right lung (Figure 1).

On ultrasound scan, the findings were indicative of a single live intrauterine fetus with an average gestational age of 36 weeks and 5 days+/- 2 weeks and four days in cephalic presentation. One week after admission, patient went into labor but refused normal vaginal delivery; hence, a full-term lower segment cesarean section under spinal anesthesia was planned on maternal request. There were no anesthetic complications and a healthy female baby of 2.6 kg body weight was delivered with an Apgar score of 9 and 10 at one and five minutes respectively. She and her baby are doing well after the delivery. 

## Discussion

Drug resistance in pulmonary TB develops either due to premature discontinuation or improper anti-tubercular treatment. In 2020, the World Health Organization (WHO) has recommended a newer, shorter oral regimen for patients with Multi-drug resistant TB [4]. This regimen is for 9-11 months as compared to the older longer regimen of 20 months. Multi-drug resistant TB can lead to a debilitating effect on the airway, lung parenchyma, chest wall, pleura, pulmonary vasculature, and mediastinum. Parenchymal involvement is more common. Cicatrization collapse or fibrosis usually occurs because of partial or complete involvement of lung parenchyma and airway. Radiologically there is an area of alveolar destruction with loss of lung volume and ipsilateral mediastinal shift [5]. These can be symptomatic or asymptomatic like our patient was asymptomatic. Pregnancy adds a burden on the already compromised respiratory system, particularly during labor. Anaesthetic complications particularly general anesthesia during cesarean section can risk the life of the mother and her baby [6].

## Conclusions

TB is a major concern in developing countries and even if a bacterial cure has been achieved, there could be short-term and long-term sequelae. Compliance with treatment with regular follow-up can minimize the post-tubercular sequelae in the reproductive age group. Pre-conceptional counseling, regular antenatal care coupled with a multidisciplinary approach is the key to successful outcomes. If appropriate and timely treatment is not taken, it can lead to serious debilitating irreversible outcomes. In this case, such an adverse outcome was unilateral post TB fibrosis which could have been a major problem for the normal pregnancy to continue. So the early diagnosis and management of TB hold much importance to prevent deleterious impact on the psychosocial and financial aspects of the patient.
